# The effect of mindfulness-based stress reduction training on fear of childbirth and psychological well-being in women experiencing premenstrual syndrome: a randomized controlled trial

**DOI:** 10.1007/s00737-026-01737-8

**Published:** 2026-06-19

**Authors:** Aysel Akbeniz, Esra Sabancı Baransel

**Affiliations:** 1 Tarsus University, Faculty of Health Sciences, Department of Psychiatric Nursing, Tarsus, Mersin, Turkey; 2 İnönü University, Faculty of Health Sciences, Department of Midwifery, Malatya, Turkey

**Keywords:** MBSR, Premenstrual syndrome, Childbirth fear, Psychological well-being

## Abstract

**Aim:**

This study aimed to evaluate the effects of a Mindfulness-Based Stress Reduction (MBSR) program on premenstrual symptom severity, pre-pregnancy fear of childbirth, and psychological well-being in women experiencing Premenstrual Syndrome (PMS).

**Materials and methods:**

This randomized controlled trial was conducted with 126 women experiencing PMS between June 2023 and November 2025 (MBSR group: *n* = 63; control group: *n* = 63). The intervention group participated in an 8-session online MBSR program, delivered twice weekly (40 min per session), while the control group received no intervention. Data were collected using the Premenstrual Syndrome Scale (PMSS), the Childbirth Fear - Prior to Pregnancy Scale (CF-PPS), and the Psychological Well-being Scale (PWBS). Between-group differences were analyzed using analysis of covariance (ANCOVA) adjusting for baseline values, and effect sizes were reported using partial eta-squared (η²). The trial was retrospectively registered (ClinicalTrials.gov, NCT06942169).

**Results:**

The MBSR intervention was associated with statistically significant improvements in all outcomes compared to the control group. Adjusted analyses indicated a significant reduction in premenstrual symptom severity (*p* < 0.001, η²=0.117) and fear of childbirth (*p* < 0.001, η²=0.125), as well as a significant increase in psychological well-being (*p* < 0.001, η²=0.285). These findings correspond to moderate effect sizes for PMSS and CF-PPS and a relatively large effect size for PWBS.

**Conclusion:**

The findings suggest that MBSR may be an effective non-pharmacological intervention for reducing premenstrual symptoms, decreasing pre-pregnancy fear of childbirth, and improving psychological well-being in women experiencing PMS. However, the results should be interpreted with caution due to the short-term nature of the assessment and the absence of long-term follow-up data.

## Introduction

Menstruation is a biological process that begins with puberty in women who are developing healthily, ensures fertility, and continues until menopause. During this process, some women experience a condition accompanied by physical and psychological symptoms in the late luteal phase of the menstrual cycle. This condition is called Premenstrual Syndrome (PMS) and is characterized by symptoms such as depressive mood, anxiety, irritability, fatigue, dizziness, changes in sleep and appetite, breast tenderness, and muscle and joint pain (Al-Shahrani et al., 2021; Arıöz and Ege 2013). PMS is not only a physiological problem; it is a multifaceted condition that can negatively affect an individual’s daily life activities, academic and professional performance, social relationships, and overall psychological well-being (Kahyaoglu Sut and Mestogullari 2016; Pinar et al. 2011).

Psychological and behavioral changes associated with PMS can lead to increased anxiety and emotional instability in the individual. This situation can lead to the emergence or increase of fear of childbirth in the pre-pregnancy period. Anxiety, low stress tolerance, and intense emotional fluctuations accompanying PMS can negatively affect an individual’s expectations and concerns about the birthing process. Studies show that women with high levels of anxiety and stress during the premenstrual period are more likely to develop fear and anxiety related to pregnancy and childbirth (Stoll and Hall 2013; Uçar and Taşhan 2018). Therefore, PMS can be considered a potential risk factor affecting psychological preparedness for childbirth and perceptions of safe childbirth. The relationship between PMS and pre-pregnancy fear of childbirth may also be explained through several cognitive-emotional mechanisms. Women experiencing PMS frequently report increased anxiety sensitivity, emotional dysregulation, catastrophizing tendencies, and intolerance of uncertainty during the premenstrual period. These psychological processes may contribute to heightened body-related fears and negative expectations regarding reproductive experiences, including pregnancy and childbirth. Previous literature has shown that anxiety sensitivity and psychological vulnerability are associated with increased fear of childbirth and anticipatory childbirth-related anxiety (Fairbrother et al. 2017). In this context, recurrent emotional and somatic distress associated with PMS may contribute to the development of negative cognitive schemas related to childbirth and reproductive health experiences.

In addition, the effects of PMS on psychological well-being are also important. Psychological well-being is a concept that holistically reflects an individual’s positive emotional state, life satisfaction, self-efficacy perception, and capacity to cope with stress. The severity of PMS symptoms can negatively affect an individual’s psychological well-being by increasing their stress and anxiety levels. PMS symptoms, particularly depressive mood, irritability, anxiety, and fatigue, can weaken an individual’s self-esteem, limit daily functioning, and reduce their quality of life (Arbabi et al. 2008).

In this context, PMS is not only a condition with physiological symptoms but also one that has statistically significant effects on psychological well-being and perceptions related to childbirth. In recent years, psychosocial interventions aimed at managing stress and anxiety have emerged as effective methods in reducing PMS symptoms and related psychological problems. Recent evidence suggests that various non-pharmacological interventions may contribute to the management of PMS symptoms and associated psychological difficulties. Exercise-based interventions have been reported to reduce negative affect, pain, and fatigue in women experiencing PMS (Li et al. 2026).Similarly, web-based progressive muscle relaxation and lifestyle interventions have demonstrated positive effects on psychological well-being, occupational performance, and quality of life in women with PMS (Karakus and Akyurek 2026). In addition, cognitive therapy-based interventions have been shown to support the management of emotional and behavioral symptoms associated with PMS (TS et al. 2023). Previous meta-analytic evidence has also suggested that psychological interventions, particularly cognitive-behavioral approaches, may contribute to reductions in anxiety, depressive symptoms, and impairment in daily functioning among women experiencing PMS (Busse et al. 2009). Moreover, emerging evidence indicates that mindfulness-based and digitally delivered interventions may improve emotional regulation, quality of life, and symptom severity in women with PMS and PMDD (Panahi and Faramarzi 2016; Askari et al. 2022; Puthusserry and Delariarte 2023). These findings suggest that behavioral, cognitive, lifestyle, and psychosocial approaches may play an important role in PMS management. Within this broader framework, MBSR may offer a holistic approach by targeting stress regulation, emotional awareness, and psychological resilience simultaneously.

Among these interventions, MBSR programs aim to increase individuals’ awareness levels, recognize automatic stress responses, strengthen emotion regulation skills, and improve psychological resilience. The positive effects of MBSR programs on depression, anxiety, stress, and psychological well-being have been supported by research conducted in various clinical and healthy populations (Kabat-Zinn and Hanh 2009; Sulosaari et al. 2022).

MBSR has the potential to both reduce premenstrual symptoms and mitigate the negative effects of PMS on fear of childbirth and psychological well-being in women experiencing PMS. The literature suggests that MBSR strengthens individuals’ psychological resources by reducing anxiety and stress levels, thus potentially having positive effects on fear of childbirth and overall psychological well-being. Therefore, systematically evaluating MBSR intervention in women experiencing PMS fills an important research gap in terms of both symptom management and support for psychological health. Given the aforementioned PMS-related symptoms, increased fear of childbirth, and negative effects on psychological well-being, researching non-pharmacological, holistic, and psychosocial interventions in women experiencing PMS is crucial.

Previous studies have demonstrated that mindfulness-based interventions, including MBSR and mindfulness-based cognitive therapy, can effectively reduce premenstrual symptoms such as anxiety, irritability, and mood disturbances (Panahi and Faramarzi 2016; Bluth et al. 2015; Şener Çetin and Şolt Kırca 2023). These studies primarily focused on symptom reduction and emotional regulation in women experiencing PMS or PMDD.

## Research hypotheses

### H1

Premenstrual syndrome scores of women experiencing PMS who participate in the MBSR program will be significantly reduced compared to the control group.

### H2

Pre-birth fear scores of women experiencing PMS who participate in the MBSR program will be significantly reduced compared to the control group.

### H3

Psychological well-being scores of women experiencing PMS who participate in the MBSR program will be significantly increased compared to the control group.

## Materials and methods

### Study design

This study was designed as a randomized-controlled study. The study procedures were approved by the Inonu University Scientific Research and Publication Ethics Committee (Decision No: 4615) and comply with the ethical guidelines outlined in the Helsinki Declaration. This study retrospectively registered Clinical Trials Protocol Registration and Results System (ClinicalTrials.gov), NCT06942169 (Date of registration: 22.01.2026). The retrospective registration was due to administrative and procedural delays at the study initiation stage and was not related to selective outcome reporting or post hoc modifications. The study protocol, outcome measures, and statistical analysis plan were finalized before participant recruitment began. No deviations from the original study protocol occurred during the conduct of the trial.

In the trial registry, the primary and secondary outcomes were described in general terms, including “premenstrual syndrome severity,” “fear of childbirth,” and “psychological well-being.” In the present manuscript, these outcomes are operationalized using validated and widely used measurement instruments, namely the PMSS, the Childbirth Fear-Prior to Pregnancy Scale (CF-PPS), and the Psychological Well-being Scale (PWBS). This reflects a clarification and specification of outcome assessment rather than a change in outcomes. No outcome switching occurred; all reported outcomes were prespecified in the trial registration, although described in less specific terms.

Similarly, the intervention was registered broadly as a MBSR program. In this manuscript, detailed information regarding the intervention content, duration, and delivery format has been provided to enhance transparency and reproducibility. No substantive changes were made to the intervention protocol after study initiation. Furthermore, the study was conducted in accordance with Good Clinical Practice guidelines. All participants signed an informed consent form before starting the study.

The study was conducted between June 2023 and November 2025 with women scoring 110 or higher on the PMSS scale. The sample size was calculated using G*Power software (version 3.1.9.2). The calculation was based on the total PMSS score reported by Arıöz and Ege, with an effect size (Cohen’s d) of 0.86 for the primary outcome (premenstrual syndrome severity). A two-tailed independent samples t-test was assumed, with a significance level of α = 0.05, statistical power of 0.95, and an allocation ratio of 1:1.

According to the power analysis, the minimum required sample size was 60 participants per group. Considering a potential attrition rate of 5%, the total sample size was increased to 126 participants (63 in each group).

The inclusion criteria for the study were: (1) being 18 years of age or older, (2) having regular menstruation (between 21 and 35 days), (3) having a total PMS scale score of 110 or higher, and (4) having no previous pregnancy, miscarriage, or birth experience. PMS eligibility was determined based on a total score of 110 or higher on the PMSS.

The exclusion criteria included: (1) the presence of any gynecological disease (e.g., abnormal uterine bleeding, fibroids, ovarian cysts), (2) the use of contraceptive pills or any regular medication, (3) having a diagnosed psychiatric disorder, (4) planning pregnancy during the study period, and (5) having previous experience with mindfulness or relaxation-based practices.

The withdrawal criteria included: (1) attending less than 80% of the MBSR sessions, (2) planning pregnancy or becoming pregnant during the intervention period, and (3) receiving any physical or psychiatric diagnosis during the study process.

All participants were eligible at baseline, and adherence was monitored throughout the intervention period. No participants met the exclusion criteria during the intervention period, and all randomized participants were included in the final analysis.

### Trial setting

This trial was conducted in Türkiye between June 2023 and November 2025 and all study procedures (baseline and post-intervention assessments and the MBSR sessions) were delivered online using Google Forms and Zoom, respectively.

### Randomization

Participants were randomly assigned to either the MBSR group or the control group using simple randomization based on computer-generated random numbers, with a 1:1 allocation ratio.

The allocation sequence was generated and managed by an independent researcher who was not involved in participant recruitment, intervention delivery, or outcome assessment. The allocation sequence was not accessible to the research team during the assignment process, ensuring allocation concealment.

**Fig. 1:**
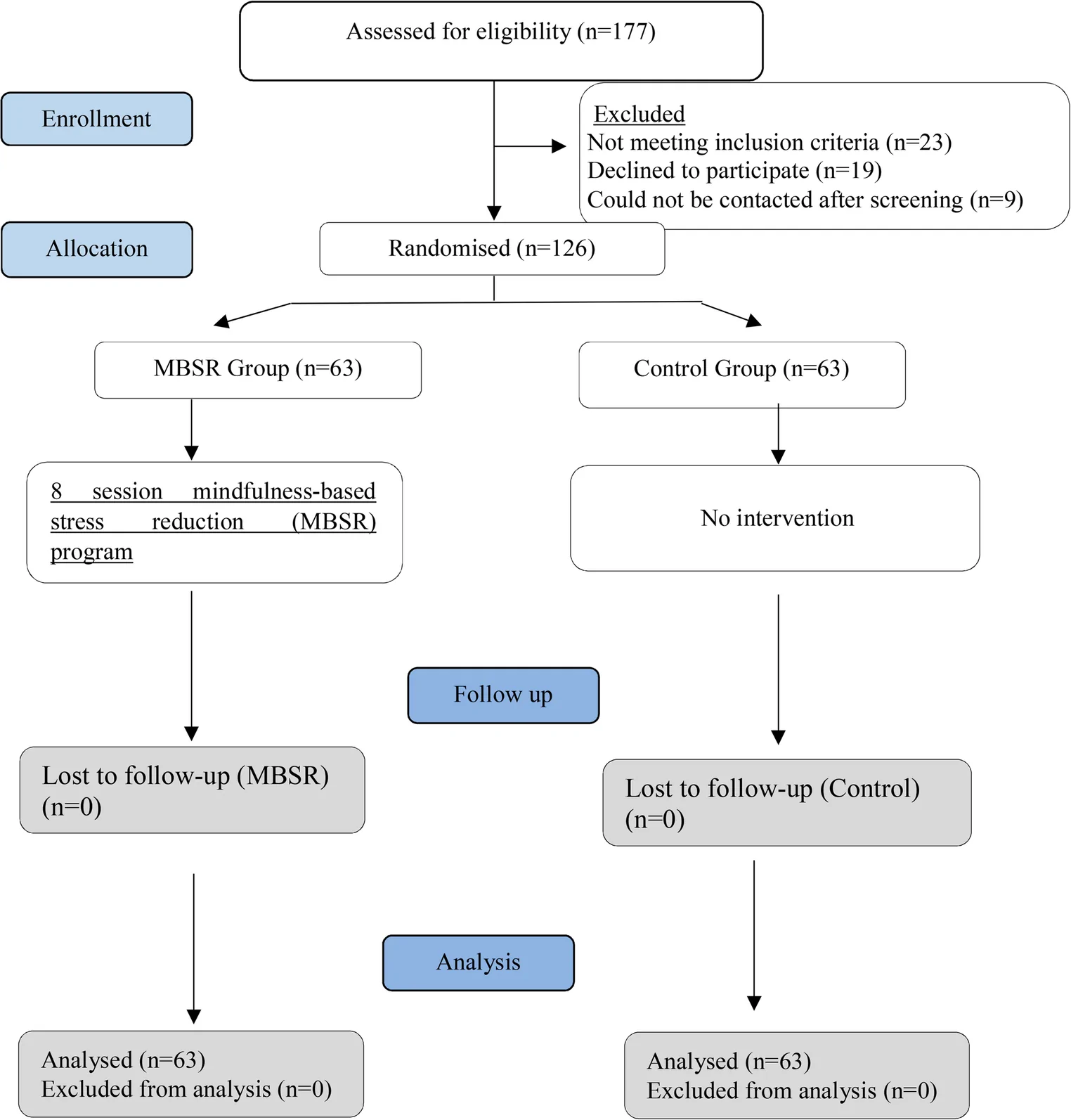
CONSORT diagram of the participants for each stage in this study

Following allocation, group assignments were necessarily known to the intervention providers due to the nature of the study design. The CONSORT flow diagram of the participants for each stage in this study is presented in Fig. 1.

### Blinding

Due to the nature of the mindfulness-based training program, participants and intervention providers were not blinded to group allocation. Outcome data were collected using self-report scales administered online.

### Interventions

The intervention applied to the experimental group was a MBSR program based on the standard framework developed by Kabat-Zinn (2003) and adapted by the researchers to suit the online delivery format and the specific characteristics of the study population. The program was delivered by a practitioner who had received formal training in mindfulness-based approaches.

The intervention was conducted via Zoom and consisted of a total of eight sessions, each lasting 40 min and delivered twice a week. The intervention was delivered online to enhance accessibility and feasibility, allowing participants to attend sessions regardless of time and location constraints. The intervention was adapted from the standard Kabat-Zinn MBSR framework to improve feasibility and participant adherence in the online setting. The shorter session duration was preferred to reduce participant burden, minimize online fatigue, and support sustained participation while maintaining the essential components of the MBSR program. Online delivery was also considered beneficial in improving participation rates and reducing potential barriers associated with face-to-face interventions. In addition, digital mindfulness-based interventions have been increasingly used in recent years and have demonstrated comparable effectiveness to in-person formats.

The program maintained the core components of MBSR, including body scan, mindful breathing, awareness exercises, and emotional regulation practices. Minor adaptations were made to session duration and structure based on the standard Kabat-Zinn MBSR framework in order to enhance feasibility, accessibility, and participant engagement in the online setting while preserving the core components of the intervention.

Following a preparatory session, participants were guided through structured mindfulness practices in each session, including introduction and awareness, body scan, raisin exercise, exiting autopilot, breath focus, sounds and thoughts meditation, emotional awareness, and mindfulness-based coping strategies. After each session, participants were assigned home practices such as body scan, breath awareness, and thought observation to support regular engagement. At the beginning of each session, participants were briefly asked about their home practice experiences and any questions related to the exercises were discussed. Participant adherence was primarily monitored through attendance records maintained during each online session. A structured session format based on the standardized MBSR framework was followed throughout the intervention to support intervention fidelity. However, adherence to home practice activities was not formally quantified.

Participants in the control group received no intervention during the study period but completed both the pre-test and post-test assessments. At the end of the study, participants in the control group were provided with an informational booklet containing the training content.

A detailed overview of the session content and home practices included in the online MBSR program is presented in Table 1.

**Table 1:** Overview of the online MBSR program

Session	Main content	Home practice
Preparatory Session	Introduction to the study process, online participation procedures, and basic information about mindfulness	None
Session 1	Introduction to mindfulness and awareness practices	Brief awareness exercises
Session 2	Body scan practice and recognizing bodily sensations	Body scan practice
Session 3	Raisin exercise and mindful attention to daily experiences	Mindful eating practice
Session 4	Exiting autopilot and developing present-moment awareness	Daily mindfulness observation
Session 5	Mindful breathing and breath-focused attention practices	Breath awareness exercises
Session 6	Sounds and thoughts meditation; observing thoughts without judgment	Thought observation exercises
Session 7	Emotional awareness and emotion regulation practices	Emotional awareness diary
Session 8	Mindfulness-based coping strategies and review of learned practices	Continued self-guided mindfulness practice

Note: The intervention was adapted from the standard Kabat-Zinn MBSR framework for online delivery while preserving the core components of mindfulness-based practice

### Data collection

In the study, women who met the inclusion criteria and volunteered to participate were first administered the PMSS. Women scoring 110 or higher on the PMSS were considered to be experiencing premenstrual syndrome. The PMSS is a valid and reliable screening instrument widely used to assess PMS symptoms. However, PMS diagnosis was not prospectively confirmed across menstrual cycles, and participant eligibility was determined based on a single self-report scale assessment. After identifying suitable participants, pre-tests were administered by the researchers via Google Forms.

Both groups were administered the Demographic Information Form, the Premenstrual Syndrome Scale, the Pre-Pregnancy Fear of Childbirth Scale, and the Psychological Well-being Scale during the pre-test phase. Following the MBSR program in the experimental group, post-test data were collected from both groups.

Throughout the research, researchers communicated online with participants, checking for missing or erroneous forms and making necessary corrections. Data were anonymized and securely stored in password-protected systems accessible only to the research team.

### Measures

The Premenstrual Syndrome Scale (PMSS), developed by Gençdoğan (2006), was used to determine premenstrual symptom levels. The Cronbach’s alpha reliability coefficient of the scale is reported as 0.75, while it was found to be 0.77 in this study.

The Childbirth Fear - Prior to Pregnancy Scale (CF-PPS), developed by Stoll and Hall (2013) and adapted into Turkish by Uçar and Taşhan (2018), was used to assess fear of childbirth. The Cronbach’s alpha was reported as 0.89 in the adaptation study, while it was found to be 0.82 in this study.

The Psychological Well-being Scale (PWBS), developed by Diener et al. (2010) and adapted into Turkish by Telef (2013), was used to evaluate psychological well-being. The Cronbach’s alpha coefficient was 0.80 in the Turkish adaptation and 0.78 in this study.

### Outcome measures

The primary outcome of this randomized controlled trial was the change in total PMSS score from baseline to post-intervention. Secondary outcomes included changes in fear of childbirth and psychological well-being, assessed using the Childbirth Fear - Prior to Pregnancy Scale (CF-PPS) and the Psychological Well-being Scale (PWBS), respectively.

All outcome measures were assessed at two time points: baseline (pre-test) and after completion of the 8 session Mindfulness-Based Stress Reduction (MBSR) program (post-test). For each outcome, changes from baseline to post-intervention were analyzed and compared between the MBSR and control groups.

### Harms

Due to the non-pharmacological nature of the intervention, adverse events were not systematically assessed using a standardized reporting form. However, participants were encouraged to report any discomfort or unintended effects during the intervention period. No adverse events were reported by participants.

### Statistical analysis

Statistical analyses were performed using IBM SPSS Statistics for Windows, Version 25.0. categorical variables were summarized as frequency and percentage, and quantitative variables as mean ± standard deviation. Descriptive statistics were used to summarize sociodemographic characteristics of the participants. In accordance with CONSORT recommendations, no inferential statistical tests were conducted to compare baseline characteristics between groups. Randomization was expected to ensure baseline comparability between groups. To evaluate the effect of the intervention, between-group comparisons were conducted using analysis of covariance (ANCOVA), adjusting for baseline values. Analyses were performed using post-test scores adjusted for pre-intervention values. Effect sizes were reported using partial eta-squared (η²). The significance level was set at *p* < 0.05. Analyses of PMS subdimensions were conducted as secondary exploratory analyses to provide a more detailed understanding of symptom changes. No formal correction for multiple comparisons was applied; therefore, these findings should be interpreted with caution.

### Protocol and statistical analysis plan

The trial protocol and statistical analysis plan are available from the corresponding author upon reasonable request.

We used the CONSORT reporting guideline (2010) and completed the CONSORT checklist to improve the transparency and completeness of trial reporting (Fig. 1).

## Results

The study was completed with a total of 126 participants (63 in the MBSR group and 63 in the control group). There were no missing outcome data, and all randomized participants completed the pre-test and post-test assessments. The sociodemographic characteristics of participants in the MBSR and control groups were comparable based on descriptive statistics (Table 2). In accordance with CONSORT recommendations, no inferential statistical tests were conducted to compare baseline characteristics.

**Table 2:** Sociodemographic characteristics of women with premenstrual syndrome

Characteristic	MBSR group (*n* = 63)		Control group (*n* = 63)	
	*n*	%	*n*	%
Age	29.76 ± 6.39		31.19 ± 9.32	
First menarche age	14.42 ± 2.63		14.01 ± 1.84	
Educational level				
High school and below	20	15.9	18	28.6
University and above	53	84.1	45	71.4
Marital status				
Married	34	54.0	32	50.8
Single	29	46.0	31	49.2
Income situation				
Income less than expenses	16	25.4	22	34.9
Income equal to expenses	40	63.5	33	52.4
Income greater than expenses	7	11.1	8	12.7
Employment status				
Employed	43	68.3	37	58.7
Unemployed	20	31.7	26	41.3

Table 3 presents the findings regarding the pre- and post-intervention comparisons of the mean PMSS, CF-PPS, and PWBS scores of women in the MBSR and control groups. The findings show that the MBSR program provided a significant improvement in all variables. PMSS scores, which measure the severity of premenstrual syndrome, decreased from 147.47 to 124.01 in the MBSR group, while there was a limited decrease from 150.65 to 145.36 in the control group; this difference is statistically significant (*p* < 0.001) and shows a moderate effect size (η² = 0.117). Similarly, the CF-PPS score, which assesses fear of childbirth, decreased from 43.82 to 35.14 in the MBSR group, while only a small decrease was recorded in the control group (from 44.49 to 43.39); this result was also found to be significant (*p* < 0.001, η² = 0.125). The PWBS score, indicating psychological well-being, significantly increased from 32.92 to 44.39 in the MBSR group, while no improvement was observed in the control group (from 34.61 to 34.38). This increase is statistically significant (*p* < 0.001) and shows a relatively large effect size (η² = 0.285). Overall, the findings suggest that the MBSR intervention is associated with statistically significant improvements in premenstrual symptoms, fear of childbirth, and psychological well-being.

**Table 3:** Comparison of outcome measures between the MBSR and control groups

Treatment group	Pretest mean (SD)	Posttest adjusted mean (SD)	*P*-value	Partial eta^2^
PMSS				
MBSR group	147.47 (41.66)	124.01^a^ (37.96)	< 0.001	0.117
Control group	150.65 (34.86)	145.36^a^ (16.95)		
CF-PPS				
MBSR group	43.82 (1.47)	35.14^a^ (1.40)	< 0.001	0.125
Control group	44.49 (1.48)	43.39^a^ (1.39)		
PWBS				
MBSR group	32.92 (13.05)	44.39^a^ (10.14)	< 0.001	0.285
Control group	34.61 (13.23)	34.38^a^ (10.15)		

*PMSS* Premenstrual Syndrome Scale, *CF-PPS* The Childbirth Fear - Prior to Pregnancy Scale, *PWBS* Psychological Well-being Scale, *SD *Standard Deviation

^a^ Adjusted post-test means were estimated using ANCOVA controlling for baseline values

**Fig. 2:**
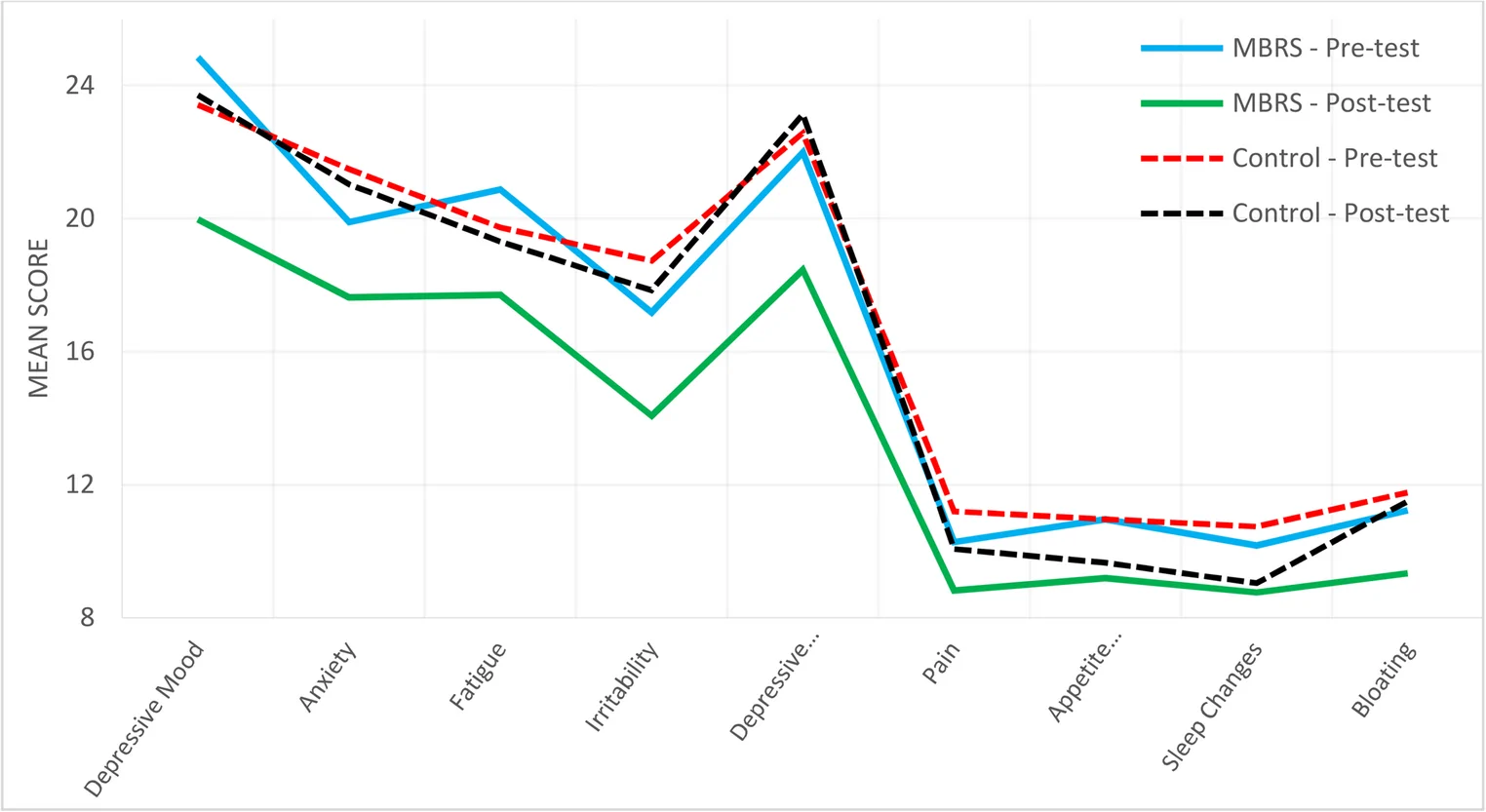
Changes in PMS subscale scores pre/post-test MBSR intervention in the study groups

Figure 2 illustrates the changes in PMS subdimensions across the study groups from baseline to post-intervention. Descriptively, greater reductions were observed in the MBSR group compared to the control group, particularly in depressive mood, anxiety, irritability, depressive thoughts, pain, and bloating. These analyses were conducted as exploratory, and the findings should be interpreted with caution due to the absence of correction for multiple comparisons. Therefore, these findings should be considered hypothesis-generating rather than confirmatory.

## Discussion

One of the key contributions of this study is the simultaneous examination of premenstrual symptoms, pre-pregnancy fear of childbirth, and psychological well-being within a single randomized controlled design. This integrated approach extends the existing literature, which has predominantly focused on symptom reduction alone. The interpretation of the findings is based on between-group comparisons adjusted for baseline values, in line with recommended statistical approaches for randomized controlled trials.

This randomized controlled trial investigated the effects of MBSR on premenstrual symptom severity, pre-pregnancy fear of childbirth, and psychological well-being in women experiencing PMS. The findings indicate that the MBSR intervention was associated with statistically significant improvements across all primary and secondary outcomes. The moderate effect sizes observed for PMSS and CF-PPS, together with the relatively large effect size for PWBS, suggest that the intervention is associated with improvements of moderate to large magnitude.

The findings of this study indicate that the MBSR intervention led to a statistically significant reduction in premenstrual symptom severity compared to the control group, based on adjusted between-group comparisons. The observed moderate effect size further supports the effectiveness of the intervention. These findings are consistent with previous studies reporting that mindfulness-based interventions reduce PMS symptoms and associated psychological distress (Panahi and Faramarzi 2016; Bluth et al. 2015; Şener Çetin and Şolt Kırca 2023).

Exploratory analyses suggested greater improvements in emotional and somatic PMS symptoms in the MBSR group, particularly in depressive mood, anxiety, irritability, depressive thoughts, pain, and bloating. However, as no correction for multiple comparisons was applied, these findings should be interpreted cautiously. This pattern may be explained by the fact that MBSR primarily targets cognitive and emotional regulation processes. Mindfulness practices are known to reduce rumination and stress reactivity while enhancing emotional awareness and self-regulation (Gu et al. 2015). However, symptoms more strongly influenced by biological and circadian mechanisms, such as sleep and appetite, may require longer-term or multimodal interventions.

Similarly, a statistically significant reduction in fear of childbirth was observed in the MBSR group compared to the control group. The moderate effect size suggests that the intervention had a meaningful impact on anticipatory childbirth-related concerns. Fear of childbirth is associated with cognitive-emotional processes such as catastrophizing, intolerance of uncertainty, and perceived loss of control. Mindfulness-based interventions may reduce perceived threat by fostering non-judgmental awareness and acceptance of internal experiences. Previous studies conducted with pregnant populations have shown that mindfulness-based programs reduce fear of childbirth and improve psychological outcomes (Van der Meulen et al. 2023; Veringa-Skiba et al. 2022). The present findings extend this evidence to the pre-pregnancy period, suggesting a potential preventive role of MBSR. One possible explanation for this finding is that women experiencing PMS may exhibit heightened sensitivity to bodily sensations, uncertainty, and anticipatory anxiety related to reproductive processes. Cognitive-emotional mechanisms such as catastrophizing, anxiety sensitivity, emotional dysregulation, and intolerance of uncertainty may contribute to the development of fear-related expectations regarding childbirth. Previous studies have shown that fear of childbirth is associated with increased anxiety vulnerability, negative cognitive schemas, and heightened emotional distress in women (Fairbrother et al. 2017; Dinç and Karataş Okyay 2021). Mindfulness-based interventions may help reduce these maladaptive cognitive-emotional responses by promoting non-judgmental awareness, emotional regulation, and acceptance of internal experiences (Şener Çetin and Şolt Kırca 2023). Therefore, the observed reduction in fear of childbirth may reflect not only decreased general anxiety levels but also improvements in cognitive-emotional processing related to reproductive health concerns.

In terms of psychological well-being, the findings indicate a statistically significant improvement in the MBSR group compared to the control group, with a relatively large effect size. However, this finding should be interpreted with caution. In particular, the use of self-reported measures and the potential influence of expectancy effects may have contributed to an overestimation of the observed effect. Previous literature has reported that mindfulness-based interventions can produce small to moderate, and in some cases large, improvements in psychological well-being (Gu et al. 2015; Koury et al. 2015). Therefore, although the findings are generally consistent with the literature, further studies incorporating objective measures and long-term follow-up are needed to confirm the robustness of these effects.

Another important consideration is that fear of childbirth was assessed in a non-pregnant population. Therefore, the findings reflect anticipatory perceptions rather than experiences related to actual pregnancy. It is possible that fear levels may differ among women who are actively planning pregnancy compared to those who are not, which was not assessed in the present study. Future research may benefit from considering pregnancy intention as a stratification variable.

Overall, the findings suggest that MBSR may be a useful psychosocial intervention for improving both symptom-related and broader psychological outcomes in women experiencing PMS. However, given the multifactorial nature of PMS, such interventions should be considered as part of a comprehensive approach that may include lifestyle modifications, medical treatment, and long-term behavioral support.

### Limitations

The study has some limitations. First, the intervention effects are based only on short-term (pre-test-post-test) measurements. The lack of long-term follow-up data limits inferences about the sustainability of the MBSR program’s effects. Future studies are recommended to evaluate the medium- and long-term effects of the intervention with follow-up measurements.

Second, the fact that the intervention was conducted online may have had a limiting effect on group interaction, therapeutic bonding, and application intensity compared to face-to-face applications. Therefore, direct generalization of the findings to face-to-face MBSR programs should be considered with caution.

Another limitation of this study is the lack of blinding of participants and intervention providers due to the nature of the MBSR program. In addition, all outcome measures were based on self-reported instruments. Therefore, expectancy effects, reporting bias, positive intervention expectations, and social desirability bias may have contributed to an overestimation of the intervention effects.

Furthermore, the absence of an active control group limits the ability to distinguish the specific effects of the MBSR intervention from non-specific factors such as attention and participation effects.

The retrospective registration of the trial represents a methodological limitation. Although the study protocol, outcome measures, and statistical analysis plan were finalized before participant recruitment began, retrospective registration may reduce transparency and should therefore be interpreted with caution.

The fact that the sample consisted of women with PMS severity above a certain threshold (PMSS ≥ 110) who voluntarily participated in the study may limit the generalizability of the findings to women with milder PMS symptoms or to different sociocultural contexts. In addition, PMS eligibility was determined using a single self-report PMSS assessment rather than prospective menstrual cycle monitoring. Since PMS symptoms may fluctuate across menstrual cycles, this approach may have increased the risk of misclassification. Therefore, the findings should be interpreted with caution. Future studies are recommended to include prospective cycle-based assessments and daily symptom diaries to improve diagnostic accuracy.

Although several reproductive and clinical variables were controlled through the inclusion and exclusion criteria, some potentially important factors such as detailed menstrual symptom characteristics, subclinical psychological symptoms, and individual differences in coping styles may still have influenced fear of childbirth, psychological well-being, and responsiveness to the MBSR intervention.

## Conclusion

In conclusion, this study suggests that MBSR may be an effective non-pharmacological approach for reducing premenstrual symptoms, decreasing pre-pregnancy fear of childbirth, and improving psychological well-being in women experiencing PMS.

However, given the short-term nature of the assessment and the absence of long-term follow-up data, these findings should be interpreted with caution. MBSR may be considered a promising supportive intervention within women’s health services, but further research with longer follow-up periods and diverse populations is needed to confirm its long-term effectiveness and clinical applicability. Future studies are recommended to incorporate objective or clinician-rated assessments, ecological momentary assessment methods, and prospective symptom diaries in order to provide a more comprehensive evaluation of intervention outcomes.

## Data Availability

The data that support the findings of this study are available from the corresponding author upon reasonable request. The data are not publicly available due to ethical restrictions and to protect the privacy and confidentiality of the participants.
